# Implementation of Chromatic Super CAZ/AVI^®^ medium for active surveillance of ceftazidime-avibactam resistance: preventing the loop from becoming a spiral

**DOI:** 10.1007/s10096-022-04480-x

**Published:** 2022-08-06

**Authors:** Gabriele Bianco, Matteo Boattini, Sara Comini, Alessio Leone, Alessandro Bondi, Teresa Zaccaria, Rossana Cavallo, Cristina Costa

**Affiliations:** 1 Microbiology and Virology Unit, University Hospital Città Della Salute E Della Scienza Di Torino, Corso Bramante 88/90, 10126 Turin, Italy; 2 Department of Public Health and Paediatrics, University of Torino, Turin, Italy

**Keywords:** Ceftazidime-avibactam resistance, Outbreak, Carbapenemase, KPC, Surveillance culture, Carbapenemase detection

## Abstract

Acquired resistance towards ceftazidime-avibactam (CAZ-AVI) is increasingly reported. Several mechanisms can be involved, but mutations in the Ω-loop region of β-lactamases are the most described. Herein, we assessed the implementation of Chromatic Super CAZ/AVI^®^ medium in rectal swab surveillance cultures in a geographic area with endemic distribution of KPC-producing *Klebsiella pneumoniae*. Routine rectal swabs collected from the intensive care unit (ICU) and non-ICU patients were screened for carbapenemase-producing Enterobacterales (CPE), carbapenem-resistant Gram-negative organisms (CR-GN) and CAZ-AVI-resistant organisms by Chromatic CRE and Super CAZ/AVI^®^ media. Among the 1839 patients screened, 146 (7.9%) were found to be colonized by one or more CPE and/or CR-GN isolates during hospitalization. Overall, among colonized patients the most common bacteria encountered were KPC-producing Enterobacterales (*n* = 60; 41.1%), carbapenem-resistant *Pseudomonas aeruginosa* (*n* = 41; 28.1%) and carbapenem-resistant *A. baumannii* (*n* = 34; 23.3%). Among patients colonized by KPC-producing Enterobacterales, thirty-five (58.3%) had CAZ-AVI-resistant strains. A 30.5% rate of faecal carriage of CAZ-AVI-resistant KPC-producing *K. pneumoniae*, substantially higher than that of susceptible isolates (2.8%), was observed in the COVID-19 ICU. Prevalence of faecal carriage of metallo-β-lactamase-producing organisms was low (0.5% and 0.2% for Enterobacterales and *P. aeruginosa*, respectively). Chromatic Super CAZ/AVI^®^ medium showed 100% sensitivity in detecting CPE or CR-GN isolates resistant to CAZ-AVI regardless of both MIC values and carbapenemase content. Specificity was 86.8%. The Chromatic Super CAZ/AVI^®^ medium might be implemented in rectal swab surveillance cultures for identification of patients carrying CAZ-AVI-resistant organisms to contain the spread of these difficult-to-treat pathogens.

**Supplementary Information:**

The online version contains supplementary material available at 10.1007/s10096-022-04480-x.

## Introduction

Ceftazidime-avibactam (CAZ-AVI) is a novel β-lactam/β-lactamase inhibitor with in vitro activity against a broad range of Gram-negative bacteria, including highly resistant strains, such as ESBL-, AmpC-, serine carbapenemase-producing Enterobacterales (CPE), and *Pseudomonas aeruginosa*, but not against metallo-β-lactamase (MBL) producers [1]. CAZ-AVI has been approved by the US Food and Drug Administration (FDA) and the European Medicines Agency (EMA) for complicated intraabdominal infections, hospital-acquired pneumonia/ventilator-associated pneumonia, and complicated urinary tract infections, as well as for the treatment of other infections due to aerobic Gram-negative organisms in adult patients with limited treatment options [2, 3].

Although it has not been long since CAZ-AVI was approved, acquired resistance is increasingly reported. Several mechanisms have been described, including amino acid substitutions in β-lactamases, alterations of ompK35/36 porins and/or overexpression of efflux pumps. Expression of KPC variants exhibiting single amino acid substitutions in the Ω-loop region (amino acid positions 164–179), and particularly the Asp179Tyr substitution, represents the most common mechanism in CAZ-AVI-resistant strains and is associated with both restoration of carbapenems susceptibility and relevant issues regarding carbapenemase phenotypic detection [4–6]. The failure to detect CAZ-AVI-resistant carbapenem-susceptible KPC variants could contribute to the escape of strains harbouring these KPC mutants from recognition by laboratorists, resulting in missed infection control practices and emergence of hospital outbreaks [7].

Recently, a selective culture medium, namely the Chromatic Super CAZ/AVI^®^ medium (Liofilchem, Roseto degli Abruzzi, Italy), was developed and evaluated for screening CAZ-AVI resistance among Gram-negative bacteria including molecularly characterized Enterobacterales and *P. aeruginosa* strains [8, 9]. The medium showed a sensitivity and specificity of 100% in detection of CAZ-AVI-resistant strains regardless of their resistance mechanisms. However, no study evaluated the implementation of Chromatic Super CAZ/AVI^®^ medium in daily clinical practice to date.

The aim of this study was to assess the performance of Chromatic Super CAZ/AVI^®^ medium by implementing it in rectal swab surveillance cultures in a geographic area with endemic distribution of KPC-producing *Klebsiella pneumoniae*.

## Material and methods

### Surveillance culture routine

The University Hospital Città della Salute e della Scienza di Torino is a tertiary-care teaching hospital in Turin, North-western Italy. Four national hospitals (San Giovanni Battista Molinette General Hospital; CTO Trauma Orthopaedic Hospital; Regina Margherita Paediatric Hospital and Sant’Anna Maternity Hospital) belong to this facility, which counts over 2.300 beds and approximately 80,000 admissions per year. Here, a proactive surveillance program to detect intestinal colonization by CPE and carbapenem-resistant Gram-negative (CR-GN) organisms among *Enterobacterales*, *P. aeruginosa* and *Acinetobacter baumannii* isolates has been adopted in acute and chronic care facilities for new admissions and for inpatients on a weekly basis. Rectal swabs are collected using the FecalSwab™ system (Copan, Brescia, Italy) and inoculated on a chromogenic screening plate (Chromatic CRE medium, Liofilchem, Roseto degli Abruzzi, Italy) by automated direct plating using the WASP^®^ instrument (Copan, Brescia, Italy). Overnight colonies are identified by MALDI-TOF MS analysis (Bruker Daltonics GmbH, Bremen, Germany), and carbapenemase production in Enterobacterales isolates is investigated by genotypic testing (Xpert Carba-R assay; Cepheid Sunnyvale, CA) and/or lateral flow immunoassay (NG-test CARBA 5; NG Biotech, Guipry, France).

### Study design

A total of 702 routine rectal swabs collected from patients admitted to COVID-19 and cardiac intensive care units (ICU) of the San Giovanni Battista Molinette General Hospital, and Pediatric Oncohematology Unit of the Regina Margherita Paediatric Hospital during a 5-month period (November 2021 to March 2022), were included in the evaluation of Chromatic Super CAZ/AVI^®^ medium. After vortexing, 10 µl of each sample was inoculated onto both Chromatic CRE (Liofilchem, Roseto degli Abruzzi, Italy) and Chromatic Super CAZ/AVI^®^ plates (Liofilchem) by automated WASP^®^ direct plating.

In addition, 132 rectal swabs collected from patients admitted to all the other departments of the above four hospitals during the study period and that tested positive to CPE or CR-GN were included. These samples, stored at a temperature of 4 °C immediately after routine culture processing, were inoculated onto SuperCAZ/AVI^®^ plates by automated WASP^®^ direct plating as soon as results were available (24–48 h after sample seeding).

After incubation at 37 °C for 18–24 h, colours of colonies grown on both media were recorded. Species identification of morphological distinct colonies was performed using MALDI-TOF MS (Bruker Daltonics GmbH, Bremen, Germany).

Suspected CPE and CR-GN isolates grown on Chromatic CRE medium and presumptive CAZ-AVI-resistant isolates grown on Chromatic Super CAZ/AVI^®^ medium were tested for CAZ-AVI, meropenem (MEM), and imipenem (IMP) susceptibility using the gradient strip method (Liofilchem Roseto degli Abruzzi, Italy), and results were interpreted according to EUCAST 2022 clinical breakpoints (https://www.eucast.org).

Carbapenemase production in Enterobacterales were investigated by lateral flow immunoassay NG-Test CARBA 5 and in case of negativity by Xpert Carba-R molecular assay (Cepheid, Sunnyvale, CA, USA). In case of negative results by both molecular and immunoassay testing, carbapenemase production was excluded by disc diffusion synergy assay [KPC, MBL and OXA-48 Confirm Kit, Rosco Diagnostica]. *P. aeruginosa* isolates resistant to CAZ-AVI were investigated for metallo-β-lactamase production by NG-Test CARBA 5 immunoassay and Xpert Carba-R assay. Carbapenem-resistant *A. baumannii* complex isolates were investigated for carbapenemase production using Amplex eazyplex^®^ SuperBug Acineto molecular assay (AmplexDiagnostics GmbH, Gars am Inn, Germany).

### Statistical analysis

Descriptive data are shown as absolute (*n*) and relative (%) frequencies for categorical data. Sensitivity, specificity, positive predictive value (PPV), and negative predictive value (NPV) of Chromatic Super CAZ/AVI^®^ medium with 95% confidence interval (95% CI) were computed using the free software MedCalc website (http://medcalc.org/).

## Results

### Screening for carbapenemase-producing Enterobacterales and carbapenem-resistant Gram-negative isolates and ceftazidime/avibactam susceptibility

A total of 351 patients admitted to COVID-19 ICU, cardiac ICU, and Paediatric Onco-haematology Unit and 1488 patients admitted to all other departments of the four hospitals of Città della Salute e della Scienza di Torino were screened for CPE and CR-GN isolates during the study period. Among these, 29 out of 72 (40.3%), 15 out of 169 (8.9%), 7 out of 110 (6.4%) and 95 out of 1488 (6.4%) patients admitted respectively to COVID-19 ICU, cardiac ICU, Paediatric Onco-haematology Unit and other departments were found to be colonized by one or more CPE and/or CR-GN isolates during the study period. Numbers of patients who tested positive on rectal swab surveillance, based on microorganisms isolated and susceptibility to CAZ-AVI, are shown in Table 1.

**Table 1:** Numbers of patients who tested positive on rectal swab surveillance in the study period based on microorganism isolated and susceptibility to ceftazidime-avibactam

		COVID-19 ICU patients *n* (%) [72 patients]	Cardiac ICU patients *n* (%) [169 patients]	Paediatric Onco-haematology Unit patients *n* (%) [110 patients]	Other Departments patients *n* (%) [1488 patients]	Total *n* (%) [1839 patients]	
KPC-producing Enterobacterales	CAZ-AVI susceptible	2 (2.8)	4 (2.4)	1 (0.9)	30 (2)		37 (2)
	CAZ-AVI resistant	22 (30.5)	1 (0.6)	0	12 (0.8)		35 (1.9)
VIM-producing Enterobacterales	CAZ-AVI susceptible	0	0	0	0		0
	CAZ-AVI resistant	0	0	2 (1.8)	4 (0.3)		6 (0.3)
NDM-producing Enterobacterales	CAZ-AVI susceptible	0	0	0	0		0
	CAZ-AVI resistant	0	0	1 (0.9)	3 (0.2)		4 (0.2)
OXA-48-like-producing Enterobacterales	CAZ-AVI susceptible	0	0	1 (0.9)	2 (0.1)		3 (0.2)
	CAZ-AVI resistant	0	0	0	0		0
KPC- and VIM-co-producing Enterobacterales	CAZ-AVI susceptible	0	0	0	0		0
	CAZ-AVI resistant	4 (5.5)	0	0	2 (0.1)		6 (0.3)
Carbapenem-resistant non-carbapenemase producing-Enterobacterales	CAZ-AVI susceptible	0	0	1 (0.9)	2 (0.1)		3 (0.2)
	CAZ-AVI resistant	0	0	0	0		0
Carbapenem-resistant *A. baumannii *complex	CAZ-AVI susceptible	0	0	0	0		0
	CAZ-AVI resistant	6 (8.3)	6 (3.5)	1 (0.9)	21 (1.4)		34 (1.8)
VIM-producing *P. aeruginosa*	CAZ-AVI susceptible	0	0	0	0		0
	CAZ-AVI resistant	0	0	0	2 (0.1)		2 (0.1)
IMP-producing *P. aeruginosa*	CAZ-AVI susceptible	0	0	0	0		0
	CAZ-AVI resistant	0	0	0	1 (0.07)		1 (0.1)
Carbapenem-resistant non-metallo-β-lactamases producing *P. aeruginosa*	CAZ-AVI susceptible	1 (1.4)	3 (1.8)	0	31 (2.1)		35 (1.9)
	CAZ-AVI resistant	0	2 (1.2)	0	1 (0.07)		3 (0.2)

Overall, among colonized patients the most common bacteria encountered were KPC-producing Enterobacterales (*n* = 60; 41.1%), carbapenem-resistant *P. aeruginosa* (*n* = 41; 28.1%) and carbapenem-resistant *A. baumannii* (*n* = 34; 23.3%). Among patients colonized by KPC-producing Enterobacterales, thirty-five (58.3%) had CAZ-AVI-resistant strains. Of these, 13 patients presented with colonization by CAZ-AVI-resistant isolates subsequent to previous colonization by susceptible isolates. In contrast, the remaining 22 patients who were admitted to COVID-19 ICU had no previous documented colonization by CAZ-AVI-susceptible KPC-producing Enterobacterales isolates. Six out of 41 (14.6%) patients who were colonized by carbapenem-resistant *P. aeruginosa* had CAZ-AVI-resistant isolates.

### Performance of Chromatic Super CAZ/AVI^®^ medium

Among 834 rectal swabs analysed, 348 samples yielded 365 Gram-negative isolates on CRE medium, of which 286 (78.3%) were CPE or CR-GN isolates. CPE isolates showed the greatest proportion (197/286, 68.9%), and *K. pneumoniae* was the most common species among KPC producers (151/153, 98.7%), KPC + VIM co-producers (9/12 75%), VIM producers (6/16, 37.5%), NDM producers (6/8, 75%) and OXA-48-like producers (4/8, 50%). Among CPE isolates, 127 were resistant to CAZ-AVI (Table 2). Eighty-six out of the 91 (94.5%) CAZ-AVI-resistant KPC-producing Enterobacterales isolates were not resistant to carbapenems (MEM MICs ranged from 0.5 to 8 mg/L; IMP MICs ranged from 0.12 to 1 mg/L) and tested negative by NG-test CARBA 5 immunoassay despite carrying *bla*_KPC_.

**Table 2:** Sensitivity, specificity, positive and negative predictive value of SuperCAZ/AVI^®^ medium

	Total isolates	CAZ-AVI resistant isolates	CAZ-AVI susceptible isolates	Sensitivity % [95% CI]	Specificity % [95% CI]	PPV % [95% CI]	NPV % [95% CI]
KPC-producing Enterobacterales^a^	153	91	62	100 [96–100%]	71 [58–81.8]	83.5 [77.4–88.2]	100
MβL-producing Enterobacterales^b^	36	36	0	100 [90.3–100]	-	100	-
OXA-48-like-producing Enterobacterales^c^	8	0	8	-	87.5 [47.3–99.7]		100
Carbapenem-resistant non-carbapenemase producing-Enterobacterales^d^	5	0	5	-	80 [28.4–99.5]		100
Carbapenem-resistant *Pseudomonas aeruginosa*	64	21	43	100 [83.9–100]	90.7 [77.9–97.4]	84 [67.4–93]	100
Carbapenem-resistant *A. baumannii* complex	20	20	0	100 [83.2–100]	-	100	-
All isolates grown on CRE medium^e^	365	168	197	100 [97.8–100]	86.8 [81.3–91.2]	86.6 [81.9–90.2]	100

^a^*K. pneumoniae*, *n* = 145; *E. coli*, *n* = 8

^b^*Enterobacter cloacae*, *n* = 18*, E. coli n* = 9; *K. pneumoniae*, *n* = 7; *Morganella morganii*, *n* = 2

^*c*^*E. coli*, *n* = 5; *K. pneumoniae*, *n* = 3

^*d*^*K. pneumoniae*, *n* = 3; *E. coli*, *n* = 2

^e^Including carbapenem-susceptible and/or non-carbapenemase-producing isolates

CR-GN isolates were *P. aeruginosa* (*n* = 64) and *A. baumannii* complex (*n* = 20), of which 32.8% and 100% were resistant to CZA-AVI, respectively (Table 2). Eighteen out of 21 CAZ-AVI-resistant *P. aeruginosa* isolates were metallo-β-lactamase producers (VIM, *n* = 16; IMP, *n* = 2) according to lateral flow immunoassay NG-Test CARBA 5 and Xpert Carba-R assay. All *A. baumannii* complex isolates tested positive to *bla*_OXA-23-like_ by Amplex eazyplex^®^ SuperBug Acineto molecular assay.

Chromatic Super CAZ/AVI^®^ medium showed 100% sensitivity [95% CI 97.8–100%] in detecting CPE or CR-GN isolates resistant to CAZ-AVI regardless of both MIC values (range: 12– ≥ 256 mg/L) and carbapenemase content (Table 2). CAZ-AVI-resistant isolates grown on Chromatic Super CAZ/AVI^®^ medium typically resulted in pink-reddish-mauve, green–blue, white-yellowish-green and white colonies for *Escherichia coli*, *K. pneumoniae*, *P. aeruginosa* and *A. baumannii*, respectively. (Supplementary Fig. 1). However, colour variations for isolates belonging to the same species were also observed (Supplementary Fig. 1-f).

Specificity and PPV of Chromatic Super CAZ/AVI^®^ medium were 86.8% [CI 95% 81.3–91.2%] and 86.6% [CI 95% 81.9–90.2%], respectively. CAZ-AVI non-resistant isolates grown on the medium (*n* = 26) showed MIC values ranging from 2 to 8 mg/L (median MIC: 4 mg/L). Rectal swabs tested positive to CPE or CR-GN isolates with CAZ-AVI MICs ≤ 2 mg/L showed no growth on Chromatic Super CAZ/AVI^®^ medium. Among the total of processed rectal swabs, four *Stenotrophomonas maltophilia* (CAZ-AVI MIC = 4 mg/L), one *Elizabethkingia meningoseptica* (CAZ-AVI MIC = 2 mg/L), one *Acinetobacter lactucae* (CAZ-AVI MIC = 4 mg/L) isolates and four *Candida albicans* isolates grew on Chromatic Super CAZ/AVI^®^ medium.

## Discussion

Active surveillance for rectal carriage of CPE and CR-GN has become a routine laboratory practice and is recommended by public-health organizations to limit the spread of these multi-drug resistant pathogens [10, 11]. Furthermore, since colonization with potential pathogens is usually a prerequisite for the development of infections, this data is of paramount importance for choosing the most appropriate empirical antimicrobial therapy in case of clinical worsening attributable to bacterial infection [12–14].

Herein, we evaluated the implementation of the Chromatic Super CAZ/AVI^®^ medium to screen CAZ-AVI-resistant isolates in rectal swabs collected from ICU and non-ICU patients. This 5-month prospective study revealed the extent of dissemination of CAZ-AVI-resistant Gram-negative organisms, especially KPC-producing *K. pneumoniae*, in a geographical area with worrisome endemic rates of CR-GN bacteria. From an epidemiological perspective, a 30.5% rate of faecal carriage of CAZ-AVI-resistant KPC-producing *K. pneumoniae* in the COVID-19 ICU, substantially higher than that of susceptible isolates (2.8%), was shown. This result agrees with the recent outbreak of CAZ-AVI-resistant KPC-33-producing *K. pneumoniae* described in the same department [7]. In contrast, among patients admitted to the Cardiac ICU, Paediatric Onco-haematology Unit and all the other departments, faecal carriage of CAZ-AVI-resistant KPC-producing Enterobacterales was substantially lower than that of CAZ-AVI-susceptible isolates. Prevalence of faecal carriage of metallo-β-lactamase-producing organisms was low (0.5% and 0.2% for Enterobacterales and *P. aeruginosa*, respectively) suggesting a low impact of this resistance mechanism on CAZ-AVI resistance in our geographical area. A high prevalence of carbapenem-resistant *A. baumannii* complex carriage (1.8%) was observed, especially in the two ICUs (8.3% and 3.5% in COVID-19 ICU and Cardiac ICU, respectively). All these isolates were found to be OXA-23-like-producers, confirming this carbapenemase as the main mechanism conferring resistance to carbapenems and CAZ-AVI [15, 16].

Although several recent studies have demonstrated the excellent activity of CAZ-AVI on international collections of non-metallo-β-lactamase-producing Gram-negative isolates [17, 18], the emergence of resistance to CAZ-AVI has been increasingly reported so that the European Centre for Disease Prevention and Control (ECDC) provided a rapid risk assessment in 2018 [5, 19]. According to the document, regular active surveillance cultures to detect CAZ-AVI-resistant Enterobacterales carriage for patients admitted to and remaining in high-risk healthcare areas are recommended in outbreak settings. To our knowledge, this is the first study evaluating the implementation of a selective medium for real-life screening of CAZ-AVI-resistant bacteria on rectal swabs. The excellent diagnostic sensitivity (100%) of Chromatic Super CAZ/AVI^®^ medium, which was preliminarily shown in studies involving full molecularly characterized bacterial strains [8, 9], has been confirmed in our active surveillance. Although specificity was 86.6%, lower than those previously reported [8, 9], integration of the Chromatic Super CAZ/AVI^®^ medium into surveillance culture diagnostic protocols for carbapenemase-resistant organisms could contribute to control dissemination of CAZ-AVI-resistant strains by rapid implementation of infection control measures. Furthermore, its use could be essential to detect CAZ-AVI-resistant Enterobacterales strains expressing KPC variants with weak carbapenemase activity. As extensively described in the literature [6, 20] and observed in this study, isolates expressing mostly KPC variants with weak carbapenemase activity and associated with CAZ-AVI resistance and low carbapenems MICs turn out to be undetectable by the main phenotypic carbapenemase detection methods including immunochromatographic assays. Since phenotypic detection methods represent the most widespread and cost-saving means of detecting carbapenemases in clinical microbiology laboratories, failure to recognize these mutated KPC–producing isolates as alert microorganisms could facilitate their spread in healthcare facilities. In addition, chromogenic selective media commonly used in active surveillance screenings for carbapenem-resistant organisms can fail to detect these strains because of low carbapenems MICs [21]. Therefore, growth of Enterobacterales on Chromatic Super CAZ/AVI^®^ medium accompanied by negativity of phenotypic carbapenemase detection tests or absence of growth on selective medium for carbapenem resistance should suggest further diagnostic investigation such as performing molecular testing for carbapenemase detection especially in areas with endemic diffusion of KPC producers. It should be noted that Chromatic Super CAZ/AVI^®^ medium represents a screening medium and its specificity in this study did not reach 90%. This suggests that this medium is not a means of assessing resistance to CAZ-AVI and that any resistance of isolates grown must be confirmed by in vitro susceptibility testing.

This study has some limitations. The performance of the medium was assessed on isolates grown on Chromatic Super CAZ/AVI^®^ and Chromatic CRE media, using as reference the CAZ-AVI susceptibility. Therefore, any CAZ-AVI-resistant isolates unable to grow on these media were excluded from the analysis. The lack of a full molecular characterization of β-lactamase gene content and cloning typing of bacterial isolates represents another limitation of our study.

In conclusion, this study provides a detailed picture of CAZ-AVI-resistant Gram-negative pathogen burden in an endemic setting posing a major challenge for the healthcare system. Given the excellent sensitivity and good specificity of Chromatic Super CAZ/AVI^®^ medium, it might be used for early identification of patients carrying CAZ-AVI-resistant organisms regardless of resistance mechanism to contain the spiral of the spread of these difficult-to-treat pathogens.

Further studies are warranted to strengthen the generalizability of our results and to extend the evaluation of Chromatic Super CAZ/AVI^®^ medium in other epidemiological contexts.

## Supplementary Information

Below is the link to the electronic supplementary material.

**Supplementary Fig. 1** Photograph of bacterial isolates grown on SuperCAZ/AVI^®^ medium: (a) *Escherichia* coli; (b) *Klebsiella pneumoniae*; (c) *Pseudomonas aeruginosa*; (d) *Acinetobacter baumannii*; (e) *Stenotrophomonas maltophilia* (f) *Enterobacter cloacae*. (PPTX 9790 KB)

## Data Availability

The authors confirm that the data supporting the findings of this study are available within the article.
