# Supplementary figures and images for: Implementation of Chromatic Super CAZ/AVI® medium for active surveillance of ceftazidime-avibactam resistance: preventing the loop from becoming a spiral
Source: Eur J Clin Microbiol Infect Dis. 2022 Aug 6;41(9):1165–71. doi: 10.1007/s10096-022-04480-x (PMC9362390; doi:10.1007/s10096-022-04480-x)

## Slide 1
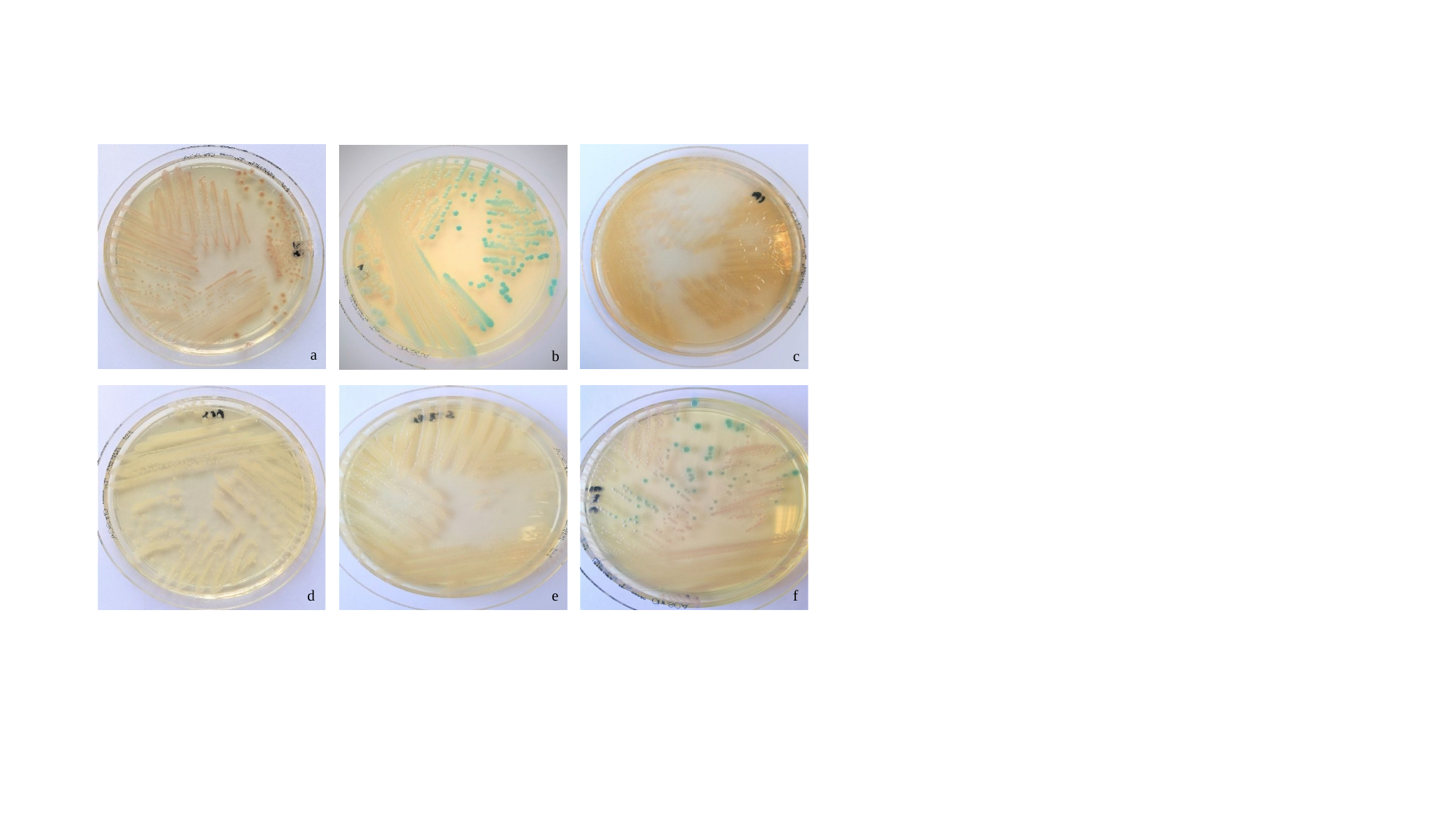

a
b
c
e
f
d

Supplement: Supplementary file 1 — Supplementary Fig. 1 Photograph of bacterial isolates grown on SuperCAZ/AVI® medium: (a) Escherichia coli; (b) Klebsiella pneumoniae; (c) Pseudomonas aeruginosa; (d) Acinetobacter baumannii; (e) Stenotrophomonas maltophilia (f) Enterobacter cloacae. (PPTX 9790 KB) [file 10096_2022_4480_MOESM1_ESM.pptx]
